# Expression and characterization of thermostable glycogen branching enzyme from *Geobacillus mahadia* Geo-05

**DOI:** 10.7717/peerj.2714

**Published:** 2016-12-06

**Authors:** Nur Syazwani Mohtar, Mohd Basyaruddin Abdul Rahman, Raja Noor Zaliha Raja Abd Rahman, Thean Chor Leow, Abu Bakar Salleh, Mohd Noor Mat Isa

**Affiliations:** 1 Faculty of Science, Universiti Putra Malaysia, Serdang, Selangor, Malaysia; 2 Malaysia Genome Institute, Kajang, Selangor, Malaysia; 3 Faculty of Biotechnology and Biomolecular Sciences, Universiti Putra Malaysia, Serdang, Selangor, Malaysia

**Keywords:** 1-4-alpha-glucan branching enzyme, His-patch thioredoxin, *Geobacillus* sp, Glycogen branching enzyme, Genome mining

## Abstract

The glycogen branching enzyme (EC 2.4.1.18), which catalyses the formation of *α*-1,6-glycosidic branch points in glycogen structure, is often used to enhance the nutritional value and quality of food and beverages. In order to be applicable in industries, enzymes that are stable and active at high temperature are much desired. Using genome mining, the nucleotide sequence of the branching enzyme gene (*glgB*) was extracted from the *Geobacillus mahadia* Geo-05 genome sequence provided by the Malaysia Genome Institute. The size of the gene is 2013 bp, and the theoretical molecular weight of the protein is 78.43 kDa. The gene sequence was then used to predict the thermostability, function and the three dimensional structure of the enzyme. The gene was cloned and overexpressed in *E. coli* to verify the predicted result experimentally. The purified enzyme was used to study the effect of temperature and pH on enzyme activity and stability, and the inhibitory effect by metal ion on enzyme activity. This thermostable glycogen branching enzyme was found to be most active at 55 °C, and the half-life at 60 °C and 70 °C was 24 h and 5 h, respectively. From this research, a thermostable glycogen branching enzyme was successfully isolated from *Geobacillus mahadia* Geo-05 by genome mining together with molecular biology technique.

## Introduction

The branching enzyme (EC 2.4.1.18) is a type of transferase that carries out the transglycosylation reaction of starch and glycogen making the structures branched out (Abad et al., 2002). Glycogen branching enzymes (GBE) are commercialised for applications in the beverage, food processing and nutraceutical industries. Studies have been done to utilize this enzyme either *in vivo* or *in vitro* in order to boost the quality of starchy food by increasing the branches in starch molecules (Kortstee et al., 1996; Kawabata et al., 2002; Kim et al., 2005; Lee et al., 2008). The branching enzyme has been used to produce cyclodextrin, a compound that is used as an ingredient in sports drinks, to enhance the taste of food and also as a spray-drying aid (Takata et al., 2010). Other than that, the branching enzyme also used in bread as an anti-staling agent, produce low viscosity and high molecular weight starch, use for paper coating and even warp sizing textile fibers to make the fibers stronger (Van der Maarel et al., 2002). Studies of GBE are also emerging with therapeutic applications; for example, against tuberculosis and glycogen branching enzyme deficiency disease (Pal et al., 2010; Garg et al., 2007; Bruno et al., 1993). The thermostable GBE is very practical in industries, but the production of this enzyme in its thermophilic host is very low. Therefore, recombinant DNA technologies, such as *Escherichia coli* cloning and expression systems, were often utilized in order to maximize enzyme production. The *E. coli* system is often preferred, as this system is easy to manipulate, capable of producing enzyme rapidly and reasonably cheap.

‘Genome mining’ is a term given to a technique that uses basic bioinformatics tools and databases to search for genes with a specific function, such as enzymes, natural products and metabolites, from genome sequences of numerous kinds of organisms (Van der Maarel et al., 2002; Ferrer, Martínez-Abarca & Golyshin, 2005; Challis, 2008). This technique exploits the readily accessible public databases that store gene and genome sequences; for example, GenBank at the National Center for Biotechnology Information (http://www.ncbi.nlm.nih.gov), the UCSC Genome Browser (http://genome.ucsc.edu) and the Ensembl Genome Browser (http://www.ensembl.org) (Corre & Challis, 2007; Schattner, 2009).

For this research, a thermophilic bacterium, *Geobacillus mahadia* Geo-05, was sampled from Sungai Klah Hot Springs, Sungkai, Perak, Malaysia at 90 °C and therefore it was postulated that this bacterium species would produce thermostable glycogen branching enzyme that is active at high temperature. The objectives of this research are to isolate and characterize glycogen branching enzyme gene (*glgB*) from *Geobacillus mahadia* Geo-05.

## Materials and Methods

### Genome mining

The genome sequence of *Geobacillus mahadia* Geo-05 used in this research was contributed by Malaysia Genome Institute. Known *glgB* nucleotide sequences from other *Geobacillus*sp. were obtained from GenBank and were used in sequence alignment softwares, local BLAST and ClustalW, to locate the position of the open reading frame (ORF) of *glgB* in the *G. mahadia* Geo-05 genome (Hall, 2010; EMBL-EBI, 2010; NCBI, 2010). *glgB* sequences of *Geobacillus* sp. obtained from GenBank that were used are *Bacillus* sp. NBRC 15315 (AB294568), *Geobacillus stearothermophilus*(M35089), *Geobacillus* sp. Y412MC10, *Geobacillus* sp. Y412MC61 (CP001794) and *Geobacillus thermodenitrificans* NG80-2. The similarity of amino acid sequence of GBE from *Geobacillus mahadia* Geo-05 compared to GBE from the other *Geobacillus* sp. are 97%, 81%, 51%, 99% and 91%, respectively.

### Microorganisms and media

The *Geobacillus mahadia* Geo-05 used in this research was contributed by the Malaysia Genome Institute (DSMZ accession number: DSM 29729). *G. mahadia* Geo-05 was grown in nutrient broth and nutrient agar (Merck). The bacteria were cultivated at 60 °C for 18 h. The genomic DNA was purified using Qiagen DNeasy^®^ Blood and Tissue Kit.

### Cloning and expression

The *glgB* from *G. mahadia* Geo-05 were amplified using polymerase chain reaction (PCR). The forward primer has additional four bases at the 5^′^ end to prepare the insert for cloning reaction into pET102/D-TOPO^®^ vector (Invitrogen). Forward primer: 5^′^–CACCATG CGA TCC AGC TTG ATT GC–3^′^; Reverse primer: 5^′^–TCA ATG ATC CGG TAC TTC CC–3^′^. Amplification process was carried out in a reaction mixture containing 20–50 ng DNA template, 0.2 µM forward and reverse primers, 0.2 mM dNTP mix, 1.2 U *Pfu* DNA polymerase and 1×*Pfu* Buffer with MgSO_4_.The genes were amplified using a thermocycler (MyCycler™, BioRad) with the temperature program of predenaturation at 95 °C for 5 min; 35 cycles of 30 s denaturation at 95 °C, 30 s annealing at 57 °C and 4 min extension at 72 °C; followed by final elongation step at 72 °C for 7 min and hold at 10 °C. Fresh PCR products were cloned into pET102/D-TOPO^®^ vector from Champion™ pET Directional TOPO^®^ Expression Kit expressed in *E. coli* BL21 Star™ (DE3).

Expression was done in 200 mL LB broth containing 100 µg/mL ampicillin in 1 L shake flask, incubated at 37 °C with 250 rpm shaking in INFORS HP (Ecotron) incubator shaker. The expression was induced with 0.75 mM IPTG when optical density A_600nm_ reached 0.5 for 8 h. After induction, cell culture was centrifuged at 12,000× g for 20 min at 4 °C.

### Protein purification

The cell pellet was resuspended in 10 mL of 50 mM sodium phosphate buffer (pH 7.0), sonicated (Branson Digital Sonifier; 2 min with 30 s lapse; amplitude: 30%) and protein aggregates was separated from soluble protein by centrifugation (12,000× g, 20 min, 4 °C). Recombinant GBE (GBE-05) (soluble protein) was purified by affinity chromatography technique using Äkta Explorer (GE Healthcare). The cleared cell lysate was loaded into 1 mL HisTrap HP column (GE Healthcare) at flow rate of 1 mL/min. The column was then washed with 20 column volume of binding buffer (20 mM sodium phosphate, 0.5 M NaCl, 30 mM imidazole, pH 7.4) and the bound enzyme was eluted with elution buffer (20 mM sodium phosphate, 0.5 M NaCl, 0.5 M imidazole, pH 7.4) by a linear gradient. Eluted protein fractions were pooled and subjected to buffer exchange using 30,000 mwco spin column (Millipore) to the buffer that was used for the assay and analysed using SDS-PAGE. SDS-PAGE (12% running gel, 6% stacking gel) was done using Laemmli’s method (Laemmli, 1970). The sample (10 µL) was loaded into the gel and run at 180 volts for 1 h. The gel was then stained with Coomassie Brilliant Blue R-250 solution. The protein content was determined by Quick StartTM Bradford protein assay (Biorad).

### Iodine stain assay

Enzyme solution in 50 mM sodium phosphate buffer, pH 7.0 (50 µl) was incubated with 50 µl of substrate at 50 °C for 30 min. The substrate was 0.1% amylose from potato (Sigma) dissolved in 50 mM sodium phosphate buffer (pH 7.0) and 10% (v/v) of DMSO. The reaction was terminated by the addition of 1 mL of iodine reagent. Iodine reagent was prepared fresh from 0.5 mL of stock solution (0.26 g of I_2_ and 2.6 g of KI in 10 mL of distilled water), 0.5 mL of 1 M HCl and diluted to 130 mL in distilled water. One unit (U) of enzyme activity was defined as the decreased of A_660nm_ reading by 1% per minute. The decreased of A_660nm_ reading represents the amylose-iodine complex (Shinohara et al., 2001).

### Enzyme characterization

The effect of temperature on GBE-05 activity was studied at temperatures from 30 °C to 80 °C with 5 °C intervals. The enzyme thermostability test was done by incubating the enzymes at 40 °C–80 °C for 24 h with 4 h intervals. After the incubation, the enzyme was immediately cooled in an ice bath prior to assay. GBE activity was assayed at 50 °C, pH 7.0. The effect of pH on GBE-05 activity was studied at pH 4–pH 10. GBE-05 activity was assayed in 50 mM acetate buffer for pH 4–6, 50 mM potassium phosphate buffer for pH 6–8, 50 mM Tris-Cl buffer for pH 8–9 and50 mM glycine-NaOH for pH 9–10. The effect of pH on GBE-05 stability was studied by incubating the enzyme in the buffers mentioned at 25 °C for 1 h. GBE activity was assayed at 50 °C, pH 7.0. To study the effect of metal ions on GBE-05 activity, GBE-05 was treated with 1 mM and 5 mM of metal ions (Mg^2+^, Ca^2+^, Fe^2+^, Mn^2+^, Zn^2+^ and Cu^2+^) for 30 min at 25 °C and immediately assayed after the treatment at 50 °C, pH 7.0.

### Nucleotide sequence accession number

The nucleotide sequence data reported in this paper are registered with the GenBank nucleotide sequence databases under accession number KC951870.

**Table 1:** Conserved regions in glycogen branching enzyme from *Geobacillus* spp., *Escherichia coli* and *Mycobacterium tuberculosis*.

	Conserved region			
	I	II	III	IV
*Geobacillus mahadia* Geo-05	HQAGLGVII**D**WVPG**H**FCK	HVDGF**R**V**D**AVAN	VLMIA**E**DSTDW	FILPFS**HD**EVV
*Geobacillus* sp. Y412MC10	HQAGIGVLL**D**WVPA**H**FAK	HIDGL**R**V**D**AVTS	ALMMA**E**ESSAW	FTLPLS**HD**EVV
*Geobacillus* sp. Y412MC61	HQAGLGVII**D**WVPG**H**FCK	HVDGF**R**V**D**AVAN	VLMIA**E**DSTDW	FILPFS**HD**EVV
*Geobacillus* sp. NBRC 15315	HQAGIGVIL**D**WVPG**H**FCK	HVDGF**R**V**D**AVAN	VLMIA**E**DSTDW	FILPFS**HD**EVV
*Bacillus stearothermophilus*	HQQGIGVIL**D**WVPG**H**FCK	HVDGF**R**V**D**AVAN	ILMIA**E**DSTDW	FILPFS**HD**EVV
*Geobacillus thermodenitrificans* NG80-2	HQAGIGVIM**D**WVPG**H**FCK	HIDGF**R**V**D**AVAN	VLMIA**E**DSTDW	FILPFS**HD**EVV
*Escherichia coli*	HAAGLNVIM**D**WVPG**H**FPT	GIDAL**R**V**D**AVAS	AVTMA**E**ESTDF	FILPFS**HD**EVV
*Mycobacterium tuberculosis*	HQAGIGVIV**D**WVPA**H**FPK	HIDGL**R**V**D**AVAS	IVTIA**E**ESTPW	YVLPLS**HD**EVV

**Notes.**

The conserved amino acids are in bold.

## Results and discussion

### Genome mining

*glgB* of *G. mahadia* Geo-05 has the size of 2013 bp that codes for 670 amino acids. The theoretical molecular weight is 78.43 kDa, predicted using the “Compute pI/Mw tool” from ExPASy Bioinformatics Resource Portal (http://web.expasy.org/compute_pi/). The four conserved regions of *α*-amylase family enzymes were determined (Table 1). Within the four conserved regions, there are seven highly conserved amino acids that have important roles in the catalysis and substrate binding. Three of the conserved residues are the catalytic residues; Asp^313^ in region II, Glu^356^ in region III and Asp^424^ in region IV. Four other conserved residues; Asp^243^ and His^248^ in region I, Arg^311^ in region II and His^423^ in region IV are responsible for substrate binding (Abad et al., 2002; Van der Maarel et al., 2003).

**Table 2:** Purification of GBE from *Geobacillus mahadia* Geo-05 using affinity chromatography.

Sample	Total protein (mg)	Total activity (u)	Specific activity (u/mg)	Purification fold	Recovery (%)
Cell extract	4.86	1314.50	270	1	100
Purified GBE	0.43	1105.28	2,598	10	84

### Protein purification

**Figure 1:**
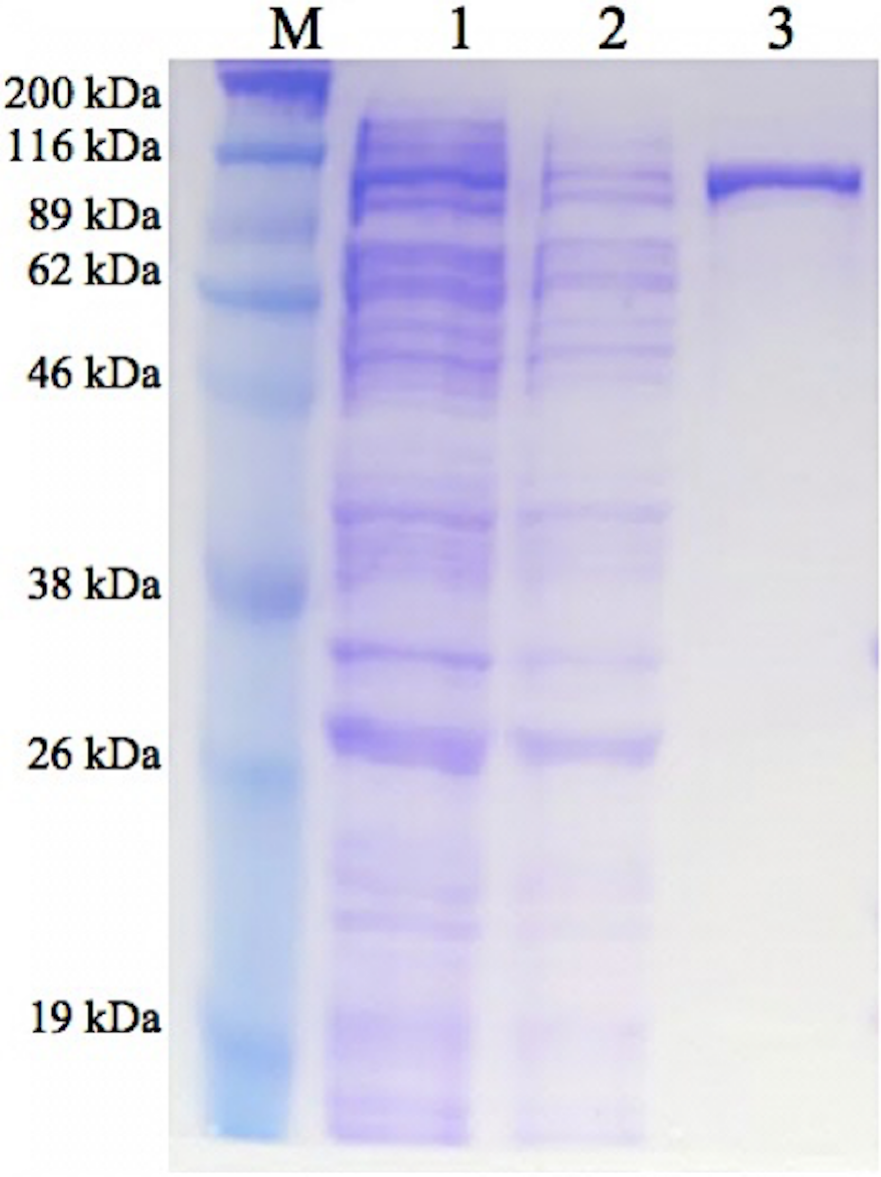
SDS-PAGE of purified enzyme. M: Broad Range Prestained Protein Marker (Nacalai). Lane 1: Crude enzyme. Lane 2: Protein in flowthrough fractions. Lane 3: Purified enzyme

GBE-05 produced by pET102/D-TOPO^®^ expression vector has His-Patch thioredoxin fused to the protein. His-Patch thioredoxin is a mutated thioredoxin that has a metal binding domain, which has been shown to have high affinity for divalent cations and therefore, the fusion protein can be purified using metal chelating resins like nickel sepharose (Lu et al., 1996). The recovery of protein obtained after the purification process was high with the enzyme activity increased by ten fold (Table 2). The SDS-PAGE result shows a single band for the purified enzyme (pooled eluted fractions) in lane 3, which means that the enzyme was successfully purified (Fig. 1). The theoretical molecular weight of GBE was 78 kDa and with the addition of His-Patch thioredoxin (13 kDa), the expected size of the recombinant protein would be 91 kDa.

### Enzyme characterization

**Figure 2:**
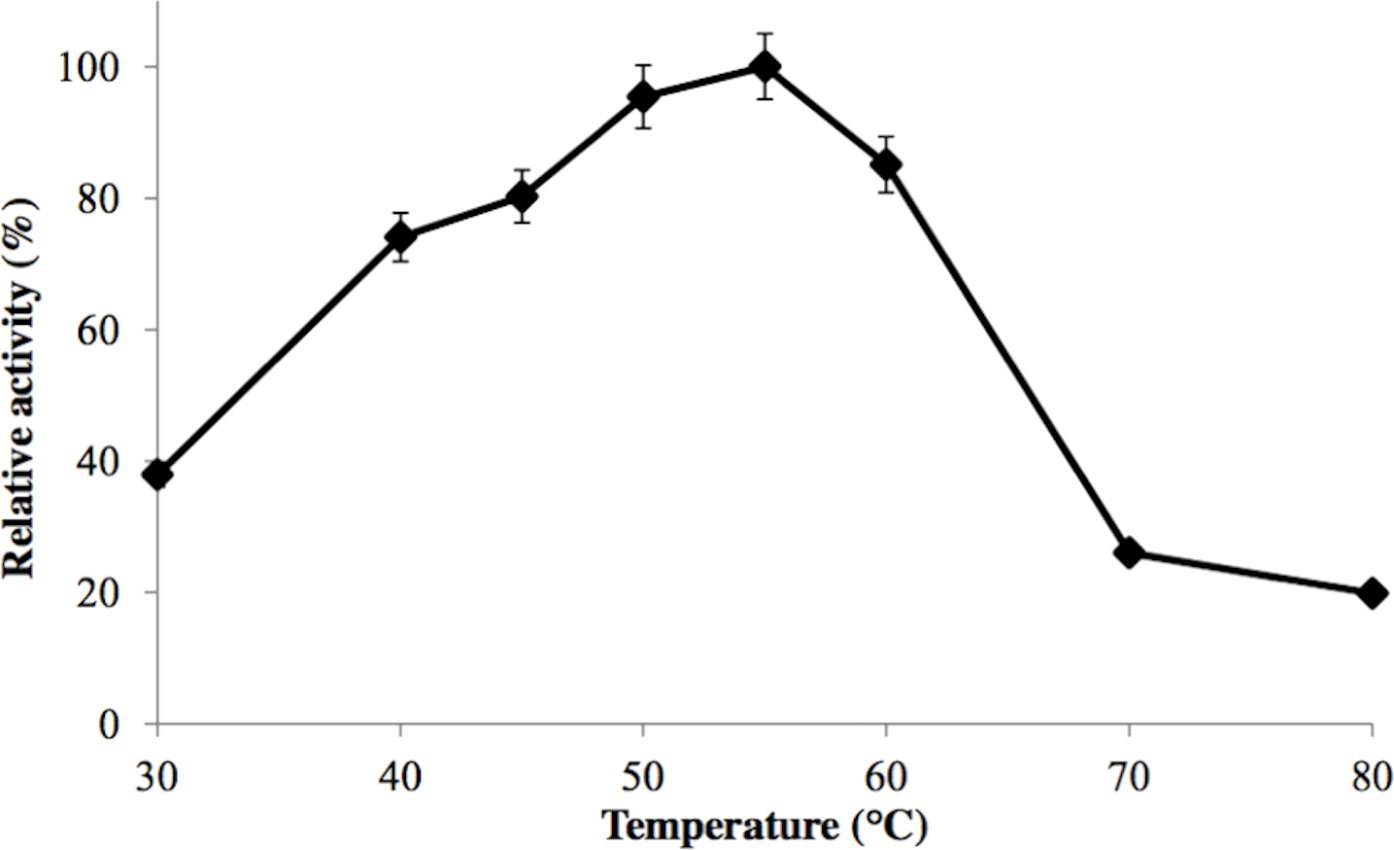
Effect of temperature on enzyme activity. GBE activity was assayed at temperature between 30 °C–80 °C. 100% of activity is 476 U/mg using iodine stain assay. Note: error bars represent means ±5% for triplicate determinations.

GBE-05 was generally active at 45 °C–60 °C and enzyme activity was highest when assayed at 55 °C (Fig. 2). This optimum temperature of GBE-05 was higher than GBEs isolated from *G. stearothermophilus* and *A. gottschalkii*, which has the optimum temperature of 50 °C (Takata et al., 1994; Thiemann et al., 2006). However, GBEs isolated from extreme thermophilic bacteria, *Rhodothermus obamensis*, *R. marinus* and *A. aeolicus* showed higher optimum temperature, that is between 65 °C–80 °C (Shinohara et al., 2001; Van der Maarel et al., 2003; Yoon et al., 2008). These bacteria produce enzymes that are active at higher temperature comparatively to their optimal growth temperatures.

**Figure 3:**
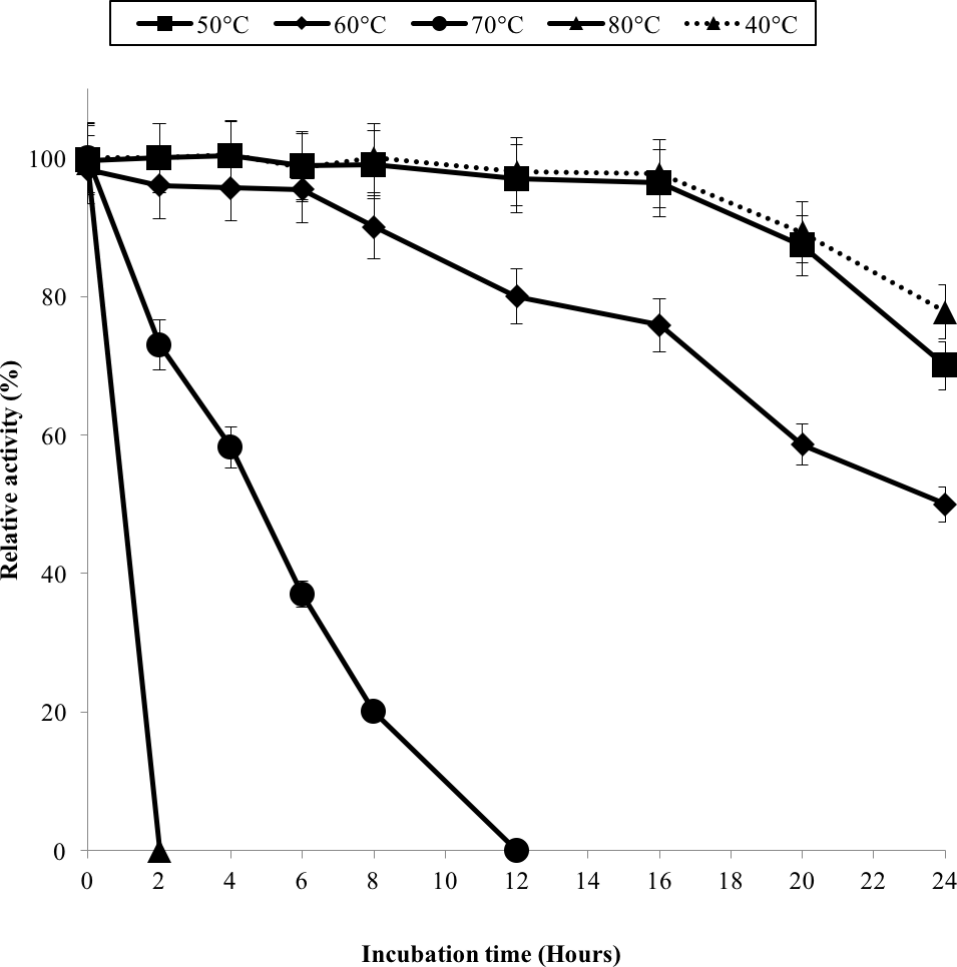
Effect of temperature on enzyme stability. GBE was incubated at 40 °C–80 °C prior to enzyme assay. Enzyme assay was done at 50 °C. 100% of activity is 793 U/mg using iodine stain assay. Note: Error bars represent means ±5% for triplicate determinations.

The half-life of the enzyme at 60 °C was 24 h while at 70 °C, 5 h (Fig. 3). GBE-05 is more stable compared to GBE from *G. stearothermophilus* that has lost 20% of enzyme activity at 60 °C in just 30 min and *A. gottschalkii* that has a half-life of only 55 min at 55 °C (Takata et al., 1994; Thiemann et al., 2006). Since GBE-05 does not have any disulphide bonds predicted, therefore the stability of this enzyme is possibly due to the high composition of aromatic amino acid residues. The thermostability of an enzyme can be presumed from its primary sequence information as there are correlations between the number of aromatic amino acids (phenylalanine, tryptophan and tyrosine), glutamine and asparagine with the thermostability (Burley & Petsko, 1985; Serrano, Bycroft & Fersht, 1991; Vieille et al., 2001; Van der Maarel et al., 2002). Enzymes with a high number of aromatic residues in combination with low number of glutamine and asparagine would show higher temperature stability. The reason behind this is that the hydrophobic interactions between the aromatic groups are responsible for the stability of a thermophilic protein, while the deamination of thermolabile amino acids (asparagine and glutamine) resulted in the inactivation of enzymes at elevated temperature (Vieille et al., 2001).

**Figure 4:**
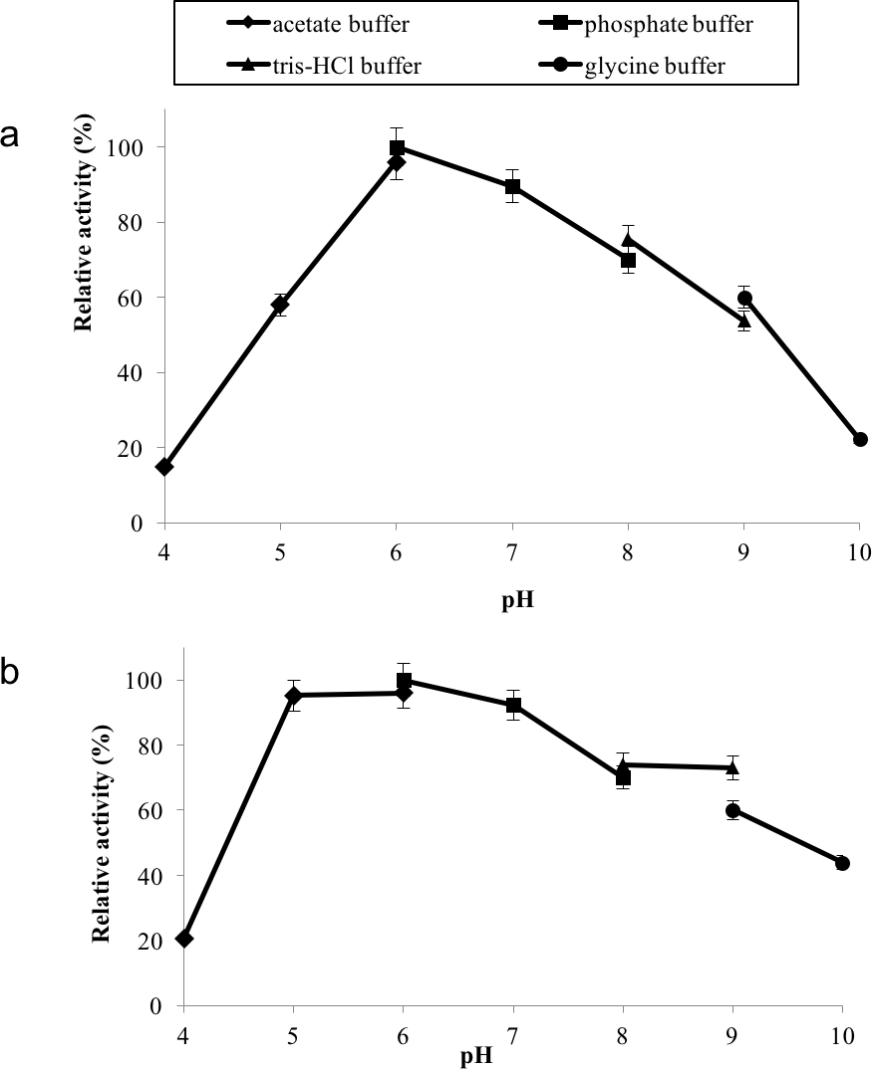
(A) Effect of pH on enzyme activity. (B) Effect of pH on enzyme stability. Note: data represents mean ± SE (*n* = 3).

GBE-05 displayed relatively high activity in broad pH range, where more than 60% of enzyme activity remained when assayed at pH 5–pH 9 (Fig. 4A), and was found to be most active at pH 6. The stability test shown that the enzyme was stable between pH 5–pH 9 where more than 50% of enzyme activity remained after the 30 min of pH treatment (Fig. 4A). It is important for GBE-05 to be active and stable in wide range of pH if this enzyme were to be applied industries.

**Figure 5:**
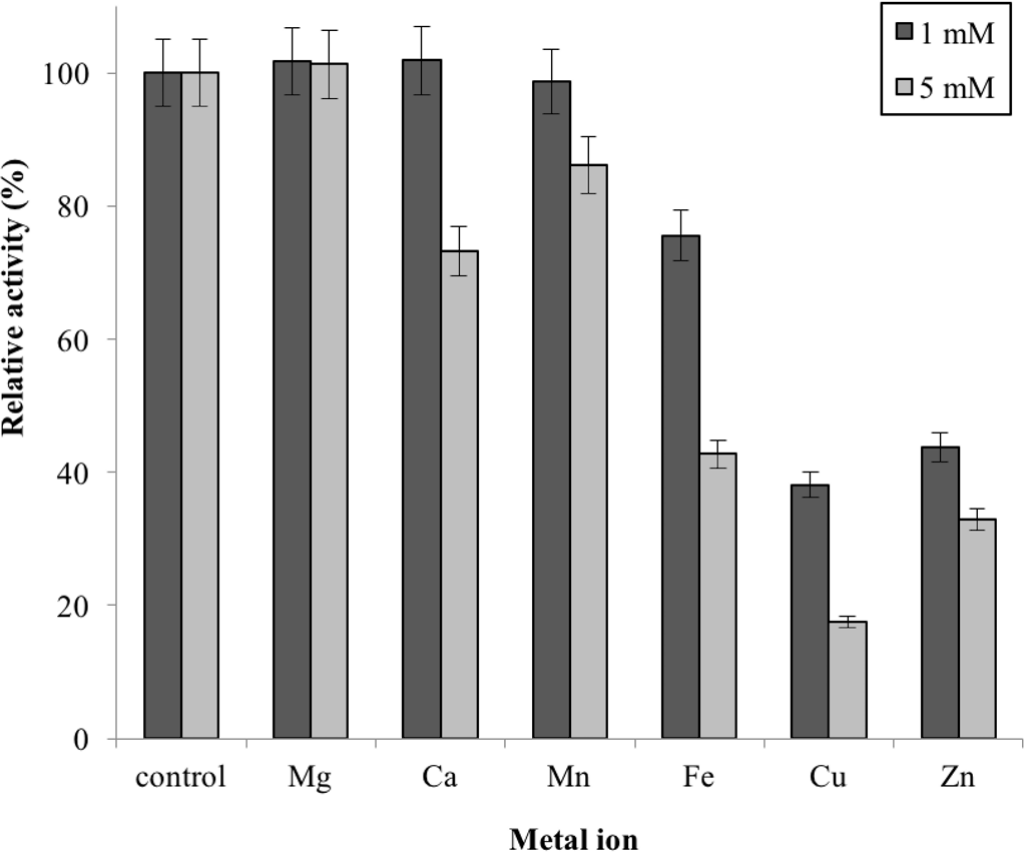
Effect of metal ion on enzyme activity. Enzyme activity was assayed with two concentrations of metal ions, 1mM and 5 mM. 100% of activity is 641 U/mg using iodine stain assay. Note: error bars represent means ±5% for triplicate determinations

Metal ions had different effects on GBE-05 activity but none of the metal ions experimented upon enhanced the enzyme activity (Fig. 5). Two alkaline earth metals of group 2 elements (Mg^2+^ and Ca^2+^) were tested to have no effect on enzyme activity. However, GBE activity was slightly lowered to 73% when the concentration of Ca^2+^ increased to 5 mM. Similar results are also observed in GBE from *M. tuberculosis* but Mg^2+^ seems to enhance the activity of GBE by 15% for *R. marinus* (Garg et al., 2007; Yoon et al., 2008). Four transition metals (Mn^2+^, Fe^2+^, Cu^2+^and Zn^2+^) were also tested out. 1 mm Mn^2+^ did not affect enzyme activity but the activity was decreased by 14% in 5 mM Mn^2+^. Mn^2+^ also showed slight inhibition on GBE activity isolated from *Anaerobranca gottschalkii* and *R. marinus* (Thiemann et al., 2006; Yoon et al., 2008). Zn^2+^ and Cu^2+^ repressed the enzyme activity as only 40% and less remained. These metal ions also appear to restrain GBE activity from other bacteria, *A. gottschalkii*, *R. marinus* and *M. tuberculosis*(Thiemann et al., 2006; Garg et al., 2007; Yoon et al., 2008). 5 mM of Fe^2+^ inhibits the enzyme by 60%, same as *R. marinus* (Yoon et al., 2008).

## Conclusions

In conclusion, GBE-05 is stable and active at high temperature and therefore is very applicable in industries. The results of genome mining and computational prediction complement the results obtained from wet laboratory experiments. The vast information on genome sequence together with latest development in structural prediction software and algorithms enables scientists to compute data from genes to protein structure and function accurately.

## Additional Information and Declarations

### Data Availability

The following information was supplied regarding data availability: GenBank. Accession number: KC951870.
